# Comparing UV and Diesel Cutaneous Damage and Evaluating the Protective Role of a Topical Antioxidant Mixture Containing Vitamin C, E and Ferulic Acid

**DOI:** 10.1111/exd.70069

**Published:** 2025-03-10

**Authors:** John Ivarsson, Alessandra Pecorelli, Anna Guiotto, Mariaurea Matias Souza, Hina Choudhary, Patricia Brieva, Francesca Ferrara, Giuseppe Valacchi

**Affiliations:** ^1^ Department of Food, Bioprocessing and Nutrition Sciences, Plants for Human Health Institute NC State University Kannapolis North Carolina USA; ^2^ Department of Animal Sciences, Plants for Human Health Institute NC State University Kannapolis North Carolina USA; ^3^ Department of Environmental and Prevention Sciences University of Ferrara Ferrara Italy; ^4^ SkinCeuticals New York New York USA; ^5^ Department of Chemical, Pharmaceuticals and Agricultural Sciences University of Ferrara Ferrara Italy; ^6^ Department of Food and Nutrition Kyung Hee University Seoul South Korea

**Keywords:** air pollution, collagen, elastin, oxinflammation, skin ageing, skin barrier

## Abstract

Cutaneous tissue is one of the main targets of outdoor stressors, and nowadays, the effect of pollution on skin conditions and premature skin ageing has been well correlated, although the exact effect that different pollutants have on the skin has not been well defined, especially when compared to other stressors. Among the air pollutants, UV radiation and particulate matter (PM) have been found among the most aggressive in terms of skin damage, inducing oxinflammatory responses, promoting degradation of extracellular matrix (ECM) components, and compromising the cutaneous defensive barrier. Topical application of technologies able to prevent oxidative damage is still one of the best approaches to protect our skin, and considering the well‐known antioxidant network, application of an antioxidant mixture is more recommended than a single compound. In the present study, human skin explants were exposed every day for 4 days to diesel particles (DEE) or to UV after the daily pre‐treatment with a topical application of a commercially available antioxidant mixture (AOX Mix), containing 15% ascorbic acid, 0.5% ferulic acid and 1% tocopherol. Oxidative stress markers such as 4‐hydroxynonenal, skin barrier proteins such as involucrin, filaggrin, claudin‐1 and desmocollin‐1, resilience markers such as elastin and tropoelastin, and the levels of Type I and Type III collagens were assessed. Topical application was able to prevent most of the damage induced by the outdoor stressors, confirming that daily protection is needed to prevent cutaneous premature ageing.

## Graphical abstract

Effect of PM versus UV on skin oxinflammatory and structural responses. Thicker arrows indicate a stronger effect. Red colours are noxious effects while green colours indicate a protective responses by topical antioxidants (AOX) application.

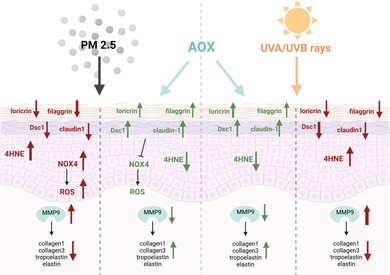

## 1 Introduction

Over recent decades, many countries around the world have taken several regulatory measures to limit their emissions of the most damaging air pollutants [1, 2]. Although there have been some improvements in air quality in the United States and European Union [3], especially following the restrictions during the Covid‐19 pandemic [4], to date, more than 90% of the world's population still lives in regions where air pollution levels are unhealthy, exceeding safe exposure limits set by the WHO Air Quality Guidelines [5].

Therefore, environmental pollution remains a major risk factor for human health, responsible for premature deaths and morbidity from respiratory and cardiovascular diseases, obesity, cancer, dementia and diabetes, contributing to 6.67 million premature deaths worldwide in 2019 [6, 7].

Particulate matter (PM_2.5_ and PM_10_), ground‐level ozone, nitrogen dioxide, sulphur dioxide and carbon monoxide represent the principal air pollutants [5]. Among these, pollution from fine particulates is considered the most dangerous for humans, as particles smaller than 2.5 μm in diameter can reach the alveoli by inhalation, cross the lung epithelium and translocate through the bloodstream into all the tissues and organs of the body [8]. Thus, PM_2.5_ can harm not only the lungs and cardiovascular system but also damage the kidneys, brain, gastrointestinal and reproductive systems [9].

As the first protective barrier of the body against pathogens and environmental insults, skin is constantly exposed to harmful pollutants that can alter its physiological homeostasis, promoting premature ageing and inflammatory skin conditions [10]. Furthermore, other exposome factors including ultraviolet radiation, tobacco smoking, poor eating habits and other lifestyle choices can accelerate skin ageing and cause dermatological problems. Interestingly, mechanistic studies revealed distinct mechanisms of action involved in skin damage response pathways to air pollutants and other exposome components [11].

Despite the debate still being open, ambient fine and ultrafine particles emitted by diesel vehicles, industrial facilities, cigarette smoke, road dust and agricultural activity could compromise skin barrier function not only by deposition on the cutaneous surface but also possibly by penetration. In fact, depending on their size and shape, PM can infiltrate the deeper layers of the skin through hair follicles or eccrine and apocrine glands as well as through an impaired skin barrier, reaching the circulation and also inducing systemic effects [12]. On the other hand, both the epidermis and dermis are well‐known targets of UV light, with the longest UVA rays able to penetrate much deeper into the papillary and reticular layers of the dermis compared to UVB and UVC, which, instead, target only the epidermis [13].

Regardless of whether or not they are able to penetrate deeply into the skin layers, both PM and UV induce oxidative stress with concomitant activation of inflammatory reactions, which initiate skin damage and, eventually, systemic conditions [14]. In this regard, understanding the individual molecular and cellular processes involved in cutaneous oxinflammatory responses to a mixture of pollutants could be critical to developing strategic approaches to prevent and/or counteract their damaging effects [8, 15, 16]. To date, few studies in the dermatological field have explored these aspects in depth, highlighting, for example, how diesel fumes, ozone and UV light, depending on their different mechanisms of action, can induce a specific pattern of alteration of microRNAs in the skin tissue [17].

Therefore, the aim of this study was to compare the noxious effects to the skin of the most damaging outdoor stressors to which we are daily exposed, such as PM and UV. Furthermore, as both PM and UV elicit oxinflammatory processes, the benefits of using a topical commercially available antioxidant mixture (AOX mix), containing 15% ascorbic acid, 0.5% ferulic acid and 1% tocopherol (C E Ferulic; SkinCeuticals, New York, NY), have also been evaluated as a strategy to limit skin damage from PM or UV.

## 2 Materials and Methods

### 2.1 Culture and Exposure of Ex Vivo Human Skin Explants

Human Caucasian skin samples were purchased from a local Cosmetic Surgery clinic (Hunstad/Kortesis/Bharti Cosmetic Surgery Clinic, Huntersville, NC, USA). Following informed consent, skin was obtained from three healthy Caucasian donors (age 35–45), undergoing routine elective abdominoplasty and skin biopsies were collected as previously reported [18]. After the overnight recovery, the medium was refreshed and the AOX mix was topically applied on the skin explants for a 24‐h pre‐treatment each day of the study. The next day, skin explants were exposed to 200 mJ/cm^2^ of UVA/UVB UV using a solar simulator (Newport, Oriel, Sol1ATM, 1600 W, Xenon Lamp, UVC & AM0 filters) or exposed for 30 min to DEE generated by a Kubota RTV‐X900 diesel engine (3‐cylinder, 4‐cycle diesel with overhead valves, 1123 cc that has 24.8 HP at 3000 rpm). Skin tissues were collected after one (Day 1) and four (Day 4) day(s) of exposure.

### 2.2 Haematoxylin and Eosin (H&E) Staining

At the indicated timepoints (Day 1 and Day 4), skin explants were collected and fixed in 10% neutral‐buffered formalin (NBF) for 24 h. Then, skin tissues were dehydrated in a gradient of alcohol and xylene and embedded in paraffin and H&E staining was performed as previously reported [18].

### 2.3 Immunohistochemistry

After deparaffinisation in xylene and rehydration in a decreasing alcohol gradient, skin tissue sections (4 μm) were subjected to antigen retrieval with a solution of 10 mM sodium citrate buffer solution (AP‐9003500; Thermo Fisher Scientific, USA) (pH 6.0) at a sub‐boiling temperature in a water bath set at 95°C for 8 min. Sections were then incubated overnight at 4°C with primary antibodies resuspended in PBS‐BSA 0.25% at the following dilutions: 4‐HNE (AB5605; Millipore Corp., USA) 1:500; MMP9 (MABT171; Millipore Corp., USA) 1:100; Filaggrin (NBP1‐87528; Novus Biologicals, USA) 1:2000; Loricrin (NBP1‐33610; Novus Biologicals, USA) 1:500; Elastin (sc‐58756; Santa Cruz Biotechnology Inc., Dallas, TX, USA) 1:50; Desmocollin (sc‐398590; Santa Cruz Biotechnology Inc., Dallas, TX, USA) 1:100; Claudin‐1 (sc‐166338; Santa Cruz Biotechnology Inc., Dallas, TX, USA) 1:200; Collagen III (sc‐514601; Santa Cruz Biotechnology Inc., Dallas, TX, USA) 1:100; Tropoelastin (MBS606788; MyBioSource, San Diego, USA) 1:50; Collagen I (ab138492; Abcam, Waltham, MA, USA) 1:200.

The day after, sections were washed and incubated with fluorochrome‐conjugated secondary antibodies (A11004 Alexa Fluor 568 and A11055 Alexa Fluor 488) for 1 h at RT. Nuclei were stained with DAPI (D1306; Invitrogen, ThermoFisher Scientific, USA). Images were acquired via epifluorescence on a Zeiss Z1 AxioObserver LSM10 confocal microscope equipped with 40× magnification. Immunofluorescence signal of at least four pictures per condition was quantified using ImageJ software [19].

Six photos were taken across the epidermis/dermis of the cutaneous paraffin section for each condition and patient at an equivalent laser gain and objective. Images were quantified using ImageJ software, using the selection tool to create an appropriate box allowing for the capture of the signal of interest (e.g., Loricrin epidermis/collagen dermis protein). The selection was saved via the ROI manager and used for each condition, allowing for equivalent areas to be quantified; a new selection was created for each marker. The selection was used to take three measurements from each photo. Measurements are expressed as the mean/average pixel intensity within the selection (raw integrated density/area of selection).

### 2.4 RNA Extraction and Quantitative Real‐Time PCR (rt‐PCR)

Tissue samples were homogenised in 1 mL of TRIzol reagent (cat. 15596018; Invitrogen, ThermoFisher Scientific, USA) with a Precellys tissue homogeniser (Bertin insturments) at 10 000 rpm at 4°C for 20 s with 30 s breaks and RNA was isolated following the manufacturer's instruction as previously reported [20]. As a reference control gene, glyceraldehyde 3‐phosphate dehydrogenase (GAPDH) was used. Table 1 summarised the primer sequences for the genes of interest. After normalisation, the fold change was determined using the $2^{-∆∆C_{T}}$ method.

**TABLE 1:** Primers sequences.

Gene	Forward sequence	Reverse sequence
LOR	GTCTGCGGAGGTGGTTCCTCT	TGCTGGGTCTGGTGGCAGATC
DSC1	CAGAGTCAAGATGGCTTCCCAG	GTTCTCAAGTCGCCAGTGTGTTG
CLDN1	GTCTTTGACTCCTTGCTGAATCTG	CACCTCATCGTCTTCCAAGCAC
ELN	GGTTGTGTCACCAGAAGCAGCT	CCGTAAGTAGGAATGCCTCCAAC
GAPDH	TCGGAGTCAACGGATTTGGT	TTCCCGTTCTCAGCCTTGAC

### 2.5 Protein Extraction and Western Blotting

Collected human skin explants were homogenised in T‐PER Tissue Protein Extraction Reagent (Thermo Fisher Scientific, USA) with 1% of phosphatase and protease inhibitor cocktails (Sigma, USA) with a Precellys tissue homogeniser (Bertin insturments) at 10 000 rpm at 4°C for 20 s with 30 s breaks, as previously described *J Cosmet Dermatol*. 2024 Apr 8. doi: https://doi.org/10.1111/jocd.16321 [21]. The following antibodies were used: 4‐HNE (AB5605; Millipore Sigma, Burlington, MA, USA), Type‐1 collagen (ab138492; Abcam, Cambridge, UK), NOX‐4 (cat. 14347‐1‐AP; ProteinTech Rosemont, IL) and as secondary antibodies (170‐6515, 170‐6516, 1721037; BioRad, USA). Detection of chemiluminescence was conducted with ChemiDoc (BioRad, USA). Bands were quantified using ImageJ software 1.53a (Java 1.8.0_172; National Institutes of Health, Bethesda, MD, USA) [22].

### 2.6 Melanin Measurements

Cutaneous melanin formation was evaluated by DermaLab skin colour probe (Cortex Technology ApS, Hadsund, Denmark). To reduce interference from pollutants and topical treatments, probe measurements were taken before daily exposure and treatment, 24 h after exposures (see Table 2). All measurements were ratioed to the baseline measurement for the biopsy to ascertain significant increases/reductions. Values were expressed as redness and pigmentation index ratio.

**TABLE 2:** Experimental design for probe measurements.

D0	D1	D2	D3	D4	D5
Baseline	Exposure 1	D1 Probes Exposure 2	D2 Probes Exposure 3	D3 Probes Exposure 4	D4 Probes

### 2.7 Statistics

Statistical analysis was performed by using GraphPad Prism 10 (Version 10.1.1 (270); GraphPad Software Inc., La Jolla, CA, USA) with an analysis of variance (one‐way or two‐way ANOVA), followed by Tukey's post hoc test for each of the variables tested. All data were expressed as means ± SEM. *p* ≤ 0.05 was considered significant in all cases.

## 3 Results

### 3.1 Experimental Conditions of DEE and UV Light Exposure Do Not Affect Skin Morphology

As depicted in Figure S1, in our experimental conditions, skin explants exposed to DEE or UV light, with or without the AOX mix, did not show any morphological changes. The H&E staining showed that the epidermis layers, starting from the basal layer until the stratum corneum (outermost layer), appear well visible and compact, with no presence of infiltrated inflammatory cells in all the different conditions. These data suggest that the adopted experimental conditions were not overly aggressive for skin tissue and suitable for the subsequent analysis in terms of OxInflammation and skin integrity.

### 3.2 Pollution‐Induced Lipid Peroxidation Is Prevented by AOX Mix Topical Application

**FIGURE 1:**
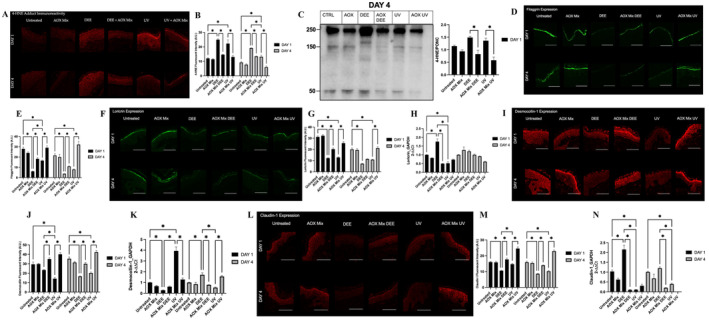
Immunofluorescence staining for 4HNE protein adducts, filaggrin, loricrin, desmocollin‐1 and claudin‐1 in human skin explants exposed to DEE or UV light and pre‐treated with AOX mix. (Panel A) A representative image of the 4HNE immunofluorescence staining(s), scale bar 100 μm. (Panel B) Depicts the signal quantification of the immunofluorescence signal performed by using ImageJ software. (Panel C) Representative image of immunoblot for 4HNE protein adduct (left panel) and bands quantification (right panel). (Panel D) A representative image of the Filaggrin immunofluorescence staining(s). (Panel E) Depicts the signal quantification of the immunofluorescence signal performed by using ImageJ software. (Panel F) A representative image of the loricrin immunofluorescence staining(s). (Panel G) Depicts the signal quantification of the immunofluorescence signal performed by using ImageJ software. (Panel H) Loricrin gene expression measured by RT‐PCR. Panel I: a representative image of the Desmocollin‐1 immunofluorescence staining(s). (Panel J) Depicts the signal quantification of the immunofluorescence signal performed by using ImageJ software. (Panel K) Desmocollin gene expression measured by RT‐PCR. (Panel L) A representative image of the claudin‐1 immunofluorescence staining(s). (Panel M) Depicts the signal quantification of the immunofluorescence signal performed by using ImageJ software. (Panel N) Claudin‐1 gene expression measured by RT‐PCR. Images were taken at 40× magnification, scale bar represents 100 μm. Data are shown as mean ± SEM and are the results of the averages of experiments conducted on three subjects, *p ≤ 0.05, **p ≤ 0.01, ***p ≤ 0.001, ****p ≤ 0.0001 by one‐way ANOVA followed by Tukey's post hoc comparison test.

Considering that the main mechanism involved in DEE and UV skin damage is represented by an altered redox homeostasis, as a proof of concept we measured the levels of 4‐hydroxynonenal (4‐HNE) protein adducts, a marker of the lipid peroxidation process and protein oxidative damage. As shown in Figure 1A, the levels of 4‐HNE protein adducts in human skin explants were mainly present in the epidermis and significantly increased upon DEE and UV exposure at both Day 1 and Day 4 (Figure 1B). Interestingly, 4HNE protein adduct levels induced by DEE were higher (35%) and persisted longer than UV, as observed at Day 4. AOX mix was able to significantly prevent the formation of 4‐HNE protein adducts for both pollutants. The data were also confirmed by Western blot, as depicted in Figure 1C, with a very evident increase of 4HNE protein adducts in DEE and UV.

### 3.3 UV‐ and DEE‐Induced Cutaneous Barrier Dysfunction in Human Skin Biopsies

Oxidative damage can lead to altered skin barrier properties; therefore, key proteins involved in keratinocytes terminal differentiation, such as loricrin and filaggrin, were analysed. These two proteins are important elements of the SC cornified envelope that represents the primary physical and biochemical barrier of the skin against the environment. As shown in Figure 1, both DEE and UV exposure significantly impaired filaggrin expression, as evidenced by the green colour (Figure 1D). DEE's effect on filaggrin levels was more evident with respect to the UV at Day 1, although both stressors significantly decreased filaggrin expression at both time points (Figure 1E). AOX mix pre‐treatment was able to prevent DEE and UV damage, preserving filaggrin expression at the control levels. As demonstrated in Figure 1F,G very similar trend was also observed for loricrin protein expression, although the AOX mix seems to be more efficient at an early time point as it concerns DEE exposure. Interestingly, as depicted in Figure 1H, DEE was able to induce a significant increase in loricrin transcript, suggesting a rescue mechanism at an early time point.

Desmocollin‐1 (Dsc1) is an isoform of desmocollin, a type of desmosomal cadherin expressed in the stratum granulosum (SG) of the epidermis. This protein is important for the structural integrity of the epidermis, namely cell–cell adhesion and keratinocyte proliferation; it helps holding neighbouring cells together, which provides strength and stability to tissues. As shown in Figure 1I, there was a significant loss of Dsc1 after UV exposure and to a less extend for DEE on Day 1, while no differences in Dsc1 levels were noticed between the two stressors at Day 4 (Figure 1I). Pre‐treatment with AOX mix was able to prevent Dsc1 loss induced by both pollutants in a significant manner (Figure 1J). In addition, UV exposure was able to clearly induce Dsc1 mRNA levels at Day 1 while the effect of DEE was more pronounced at Day 4 (Figure 1K), confirming a different way of action of the 2 pollutants and the Transcription‐translation feedback loop. Together with Dsc1, also claudin‐1 has a key role in maintain skin barrier functions. As observed for Dsc1, claudin‐1 was also significantly affected by the exposure to DEE and UV (Figure 1L), although its decrease was more pronounced at Day 4 with circa 40% loss after the different pollution exposures (Figure 1M). Pre‐treatment with AOX mix clearly prevented claudin‐1 loss and in some cases was able to further stimulate its expression over the baseline of the control. Of note, at the transcriptional levels, claudin‐1 was induced only by DEE while UV did not show any modulation at the chosen time points (Figure 1N).

### 3.4 AOX Mix Topical Application Is Able to Counteract DEE‐ and UV‐Induced NOX4 MMP9 Expression

**FIGURE 2:**
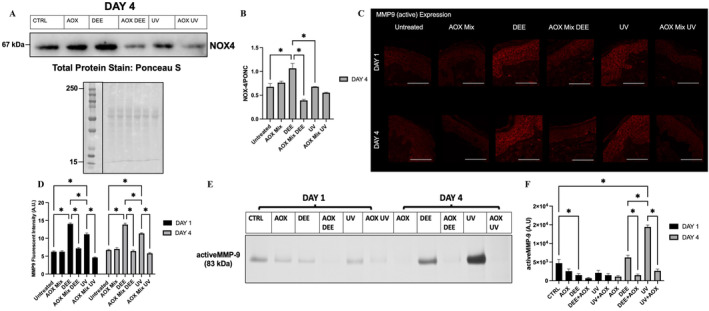
(Panel A) Representative immunoblotting for NOX‐4 protein in human skin explants exposed to indicated pollutants and pre‐treated with AOX mix after the indicated timepoint, bottom panel Ponceau Staining for loading control. (Panel B) Depicts the signal quantification of the immunofluorescence signal performed by using ImageJ software. (Panel C) A representative image of the MMP9 immunofluorescence staining(s) in human skin explants exposed to the indicated pollutants and pre‐treated with AOX mix. (Panel D) Depicts the signal quantification of the immunofluorescence signal performed by using ImageJ software. (Panel E) Representative zymogram image. (Panel F) Quantification of the zymography signal. Images were taken at 40× magnification, scale bar represents 100 μm. Data are shown as mean ± SEM and the results of the averages of experiments conducted on three subjects, *p ≤ 0.05, **p ≤ 0.01, ***p ≤ 0.001, ****p ≤ 0.0001 by one‐way ANOVA followed by Tukey's post hoc comparison test.

The ability of pollution to induce oxidative stress can be mediated by the activation of endogenous sources of ROS. Among those, there are the NADPH oxidases (Nox) enzymes. Nox4 is constitutively active, and Nox4‐dependent ROS generation is mainly regulated by its expression level. As shown in Figure 2A,B, at Day 4, there was a significant increase in Nox4 protein levels after both the pollutants exposure, and AOX mix was able to prevent the Nox4 increment by DEE and UV. An unbalanced redox status can lead to an oxidative stress condition, and this can then promote the activation of metalloproteinases (MMPs) such as MMP9, a proteolytic enzyme implicated in the degradation of proteins belonging to the extracellular matrix (ECM) such as collagen and elastin resulting in skin structural damage and premature ageing.

As depicted in Figure 2C,D, skin explants exposed to DEE or UV displayed a significant increase in the active form of MMP9 expression levels compared to control tissues, confirming that both DEE and UV could promote the initial activation of the proteolytic enzyme and be the cause of premature ageing. Notably, AOX mix pre‐treatment could restore the levels of MMP9 to basal condition in skin tissues exposed to DEE or UV. These data were also confirmed with a more quantitative method, the zymography (Figure 2E,F), which demonstrates the increase of MMP9 activation was more evident for DEE and UV at Day 4.

### 3.5 AOX Mix Prevents DEE‐ and UV‐Induced Loss of Extracellular Matrix Components Collagen and Elastin

Collagen and elastin are the main proteins involved in skin resilience. Collagen fibres are predominately responsible for providing the skin with tensile strength, while elastin provides elasticity to the skin. There are several types of collagens; however, collagen‐1 (Col 1) and collagen‐3 (Col 3) are the main types of collagens that comprise the extracellular matrix (ECM). The evaluation of the expression of both proteins is critical to demonstrate the damage caused by outdoor stressors. As previously mentioned, elastin is the other key fibre in the ECM; although not as prevalent as collagen, elastin fibres are significant due to their elasticity, allowing for the stretching and contracting of skin. Tropoelastin is the soluble monomer precursor to elastin, whose cross‐linking eventually forms mature elastic fibres. UV exposure has been associated with the degradation of ECM components, promoting a premature skin ageing process [23].

**FIGURE 3:**
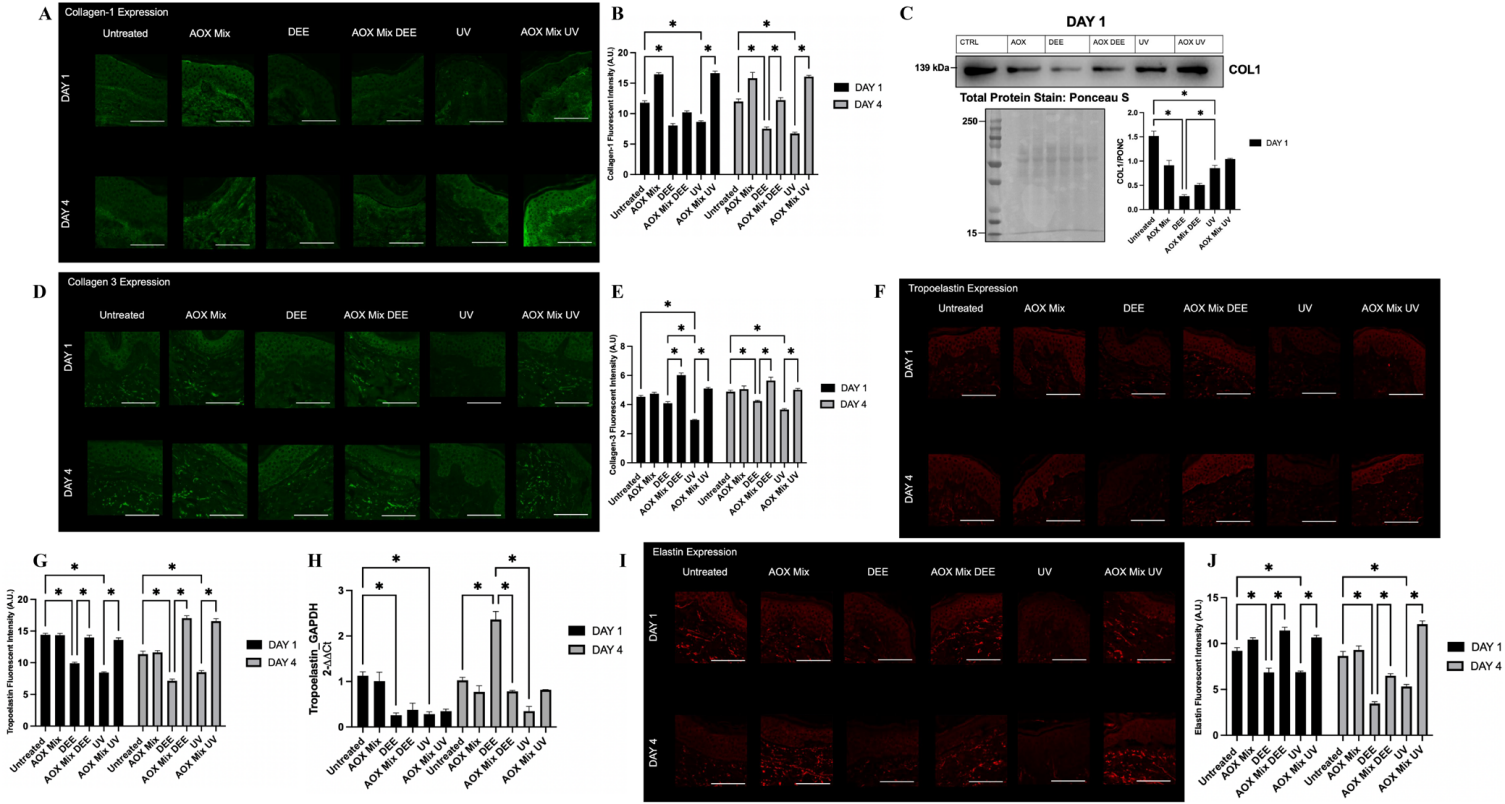
Immunofluorescence stainings for Collagen‐1, Collagen‐3, tropoelastin and elastin in human skin explants exposed to the indicated pollutants and pre‐treated with AOX mix. (Panel A) A representative image of the Collagen‐1 immunofluorescence staining(s). (Panel B) Depicts the signal quantification of the immunofluorescence signal performed by using ImageJ software. (Panel C) Representative image of immunoblot for Collagen‐1 (top panel) and quantification of the bands (lower right panel). Loading control on the bottom panel (Ponceau Staining). (Panel D) A representative image of the Collagen‐3 immunofluorescence staining(s). (Panel E) Depicts the signal quantification of the immunofluorescence signal performed by using ImageJ software. (Panel F) A representative image of the Tropoelastin immunofluorescence staining(s). (Panel G) Depicts the signal quantification of the immunofluorescence signal performed by using ImageJ software. (Panel H) Tropoelastin gene expression measured by RT‐PCR. (Panel I) A representative image of the Elastin immunofluorescence staining(s). (Panel J) Depicts the signal quantification of the immunofluorescence signal performed by using ImageJ software. Images were taken at 40× magnification, scale bar represents 100 μm. Data are shown as mean ± SEM and the results of the averages of experiments conducted on three subjects, *p ≤ 0.05, **p ≤ 0.01, ***p ≤ 0.001, ****p ≤ 0.0001 by one‐way ANOVA followed by Tukey's post hoc comparison test.

Accordingly, to the increased expression of MMP9 active form after the pollutants exposure (Figure 2C–F), a significant decrease in Col 1 (Figure 3A–C) and Col 3 (Figure 3D,E) was observed in skin tissues exposed to DEE and UV compared to the untreated control tissues. In both cases, AOX mix was able to prevent the loss of Collagens except at Day 1 for DEE exposure.

Tropoelastin (Figure 3F,G) and elastin (Figure 3I,J) protein expressions were also significantly decreased after DEE or UV exposure. The pre‐treatment of the skin biopsies with AOX mix was able to counteract the pollutants effect for both tropoelastin and elastin. AOX mix pre‐treatment was able to significantly prevent the DEE and UV‐induced loss of elastin at both time points. Of note, the AOX mix was not only able to prevent the loss of tropoelastin, but it was able to further upregulate its expression in respect to the control. In addition, tropoelastin mRNA was clearly upregulated after 4 days of DEE exposure, suggesting again transcription‐translation feedback (Figure 3H).

### 3.6 The Cutaneous Induction of Redness and Pigmentation by DEE and UV Is Prevented by AOX Mix Topical Application

**FIGURE 4:**
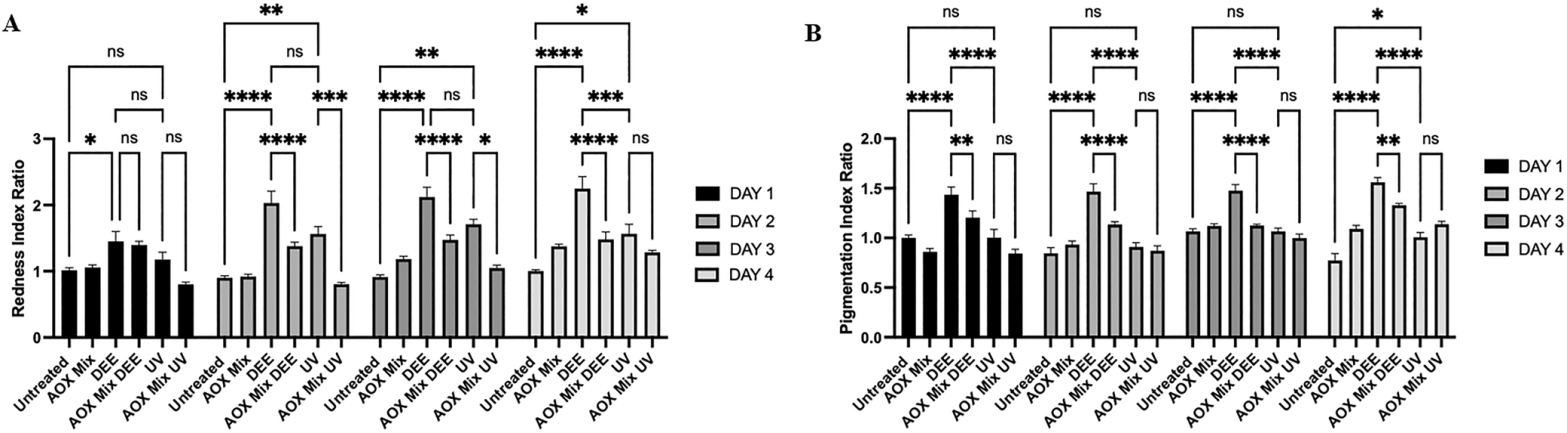
Cutaneous erythema and pigmentation measured via DermaLab Probes in human skin explants exposed to indicated pollutants with or without AOX mix pre‐treatment. Redness index and pigmentation index values are ratioed to initial baseline measurement. Data are shown as mean ± SEM and the results of the averages of 3 independent experiments conducted on three different subjects, *p ≤ 0.05, **p ≤ 0.01, ***p ≤ 0.001, ****p ≤ 0.0001 by one‐way ANOVA followed by Tukey's post hoc comparison test.

As depicted in Figure 4A, we noted an increasing erythema after DEE and UV exposure already at Day 1, which persisted for the whole 4 days. At Day 2, the redness of the skin exposed to DEE increased by twofold compared to the control and did not decrease during the experiments. AOX mix was able to counteract this effect from Day 2 until Day 4. In addition, as shown in Figure 4B, the samples exposed to DEE showed an increasing pigmentation of circa 30% compared to the control, and this was once more prevented by the topical application of the AOX mix.

## 4 Discussion

In recent years, some areas and cities around the planet have been experiencing a rising phase in levels of ambient air pollution, with severe PM_2.5_ pollution emerging as a major threat to human health [24, 25]. In addition to the well‐known effects on the cardiovascular system and lungs, there is strong evidence that external environmental aggressors, including major pollutants such as fine particulates and physical insults such as UV light, combined with internal factors, can have a damaging impact on the skin, leading to premature ageing and cutaneous conditions [26].

Therefore, in view of the worsening environmental quality, research interest in how ambient challenges exert deleterious effects on the cutaneous tissue has become popular in recent decades. In fact, a search on PubMed by typing keywords such as ‘pollution and skin’ retrieves around 8000 articles on this topic with a substantial increase in number of publications in the last 5 years. Furthermore, epidemiological studies have well‐established the direct association between exposure to poor air quality and an increase in the appearance of skin ageing as well as an aggravation of skin disorders [10, 11, 27, 28], supporting the interest in this research field.

Along with the eyes, lungs, brain and gastrointestinal tract, due to its peculiar location, the skin is a biological shield against ambient insults and, at the same time, a primary target of their adverse effects. This increased interest is related not only to the proven and well‐established worsening of air quality but also to the evidence of a direct correlation between skin conditions and air pollution exposure [29, 30]. In this context, to adopt specific actions to protect skin health and delay the signs of early ageing, it is crucial to elucidate the mechanism of action behind the damaging properties of the environmental stressors. Considering that PM and UV light are among the most harmful aggressors to which our skin is daily exposed, in the present study we were interested in understanding the specific tissue responses against the exposure to those stressors that have been shown to have different ways of action [8, 10, 13, 18, 26, 31].

Particle pollution is a complex mixture of solids and liquid particles containing dust, soil, pollen and mould spores, ash, smoke, but also organic chemicals and metals. As both a physical and chemical contaminant, PM can have a dual pattern of action. Fine particles can penetrate deep into the skin through its appendages such as hair follicles, sebaceous glands and sweat glands, disturbing its barrier function and thus cross more easily a damaged skin barrier [8, 32]. If not penetrating the skin, inorganic and organic chemical components of particles, including metals and polycyclic aromatic hydrocarbons (PAHs), can be absorbed through the skin and generate ROS, both catalysing the Fenton reaction and affecting mitochondrial function, respectively (Pardo et al. 2020). In fact, the main processes associated with PM‐induced skin disorders are an alteration of physiological redox homeostasis in conjunction with the activation of abnormal inflammatory responses [8, 32, 33].

Solar UV radiation shows the ability to penetrate the epidermis and even the dermis in a wavelength‐dependent manner. The relatively longer wavelength UVA (*λ* = 320–450 nm) which, unlike UVB, is not filtered by the stratospheric ozone layer, represents approximately 95% of the UV radiation reaching our skin and is able to penetrate more deeply than UVB into the dermis and the subcutaneous tissue [31, 34]. Although UVB is mostly filtered by Earth's atmosphere, these medium wavelengths have slightly higher energy than UVA and are almost entirely absorbed by the epidermal layer [31, 34]. Furthermore, cellular chromophores including, but not limited to, DNA, urocanic acid, aromatic amino acids, carotenoids, reduced nicotinamide adenine dinucleotide (NADH) and collagen, elastin, can show different abilities in absorbing UV bands depending on their chemical characteristics. In the stratum corneum, trans‐urocanic acid (trans‐UCA), a degradation product of histidine, acts to absorb UVA radiation which, however, leads to the formation of singlet oxygen [35]. Moreover, the UVA‐stimulated ROS production is enhanced by the release of free iron or labile iron (ferric ions) from ferritin through lysosomal proteolysis in keratinocytes and fibroblasts. By fuelling the Fenton reaction, free iron further promotes UVA‐induced ROS accumulation [13]. Similarly, UVB radiation, which is absorbed by amino acids such as tyrosine, phenylalanine and tryptophan, as well as other cellular compounds including NADH, quinones, flavines, porphyrins, 7‐dehydrocholesterol and urocanic acid, contributes to generating ROS [35].

It is therefore clear that both ambient stressors, PM and UV light, can induce cutaneous oxidative stress damage, albeit activating different cascades of complex events. Here, we confirmed this detrimental effect of PM and UV by detecting high 4HNE‐protein adducts, a very reactive and toxic aldehyde that derives from the oxidation of ω‐6 polyunsaturated fatty acids (PUFAs) [36, 37]. 4HNE‐protein adduct formation was observed in the cutaneous tissue after exposure to O_3_, PM and CS [37]. Our study showed that both UV and DEE were able to increase the levels of 4HNE protein adducts at Day 1. However, at Day 4, the effect of UV was not as evident. This trend can be explained by the different mechanisms of action of the two pollutants. Indeed, the UV effect is faster due to its ability to penetrate the skin, while PM can be absorbed over time, continuing its oxidative damage for up to 4 days. The transition metal constituents of PM (Fe, Zn, Ni, etc.) are believed to undergo Fenton or Fenton‐like reactions, generating ROS. In addition, as previously mentioned, highly lipophilic PAHs can cross the cell membrane and localise to the mitochondria, promoting the generation of mitochondrial‐produced ROS [33]. Therefore, the mechanism by which PM is able to induce the production of 4HNE is less direct but more long‐lasting. This may explain the persistently higher levels of 4HNE protein adducts on Day 4 after skin exposure to DEE.

In our experimental conditions, the involvement of an oxidative stress mechanism following the pollution exposure was indirectly confirmed by the ability of the antioxidant mix to prevent the damage induced by both UV and DEE. It should be mentioned that the commercially available antioxidant mix used in this study has already been proven to prevent UV‐induced erythema, although this effect was studied only in the case of acute UV injury [38, 39, 40]. Our study, however, has shown that this antioxidant mix offers prolonged cutaneous protection to the chronic damage of UV rays over a period of 4 days. These results are easily translated to real life, since we (and others) have already demonstrated the reliability of the skin explant model [41, 42, 43, 44, 45, 46]. It should be mentioned that skin explants do not allow evaluation of the effects of pollutants and the protective properties of topical applications for long periods since, after five days of culture, the ex vivo skin explants could start to show signs of decreased viability and compromised tissue integrity, affecting the accuracy of the results [42].

ROS tissue levels can have an exogenous and endogenous source. NOX4 enzymes catalyse the production of superoxide through reduction in NADPH. Via the generation of ROS, NOX4 mediates diverse functions such as host defence and inflammation, posttranslational processing of proteins, cellular signalling and regulation of gene expression. However, NOX4 also contributes to a wide range of skin proinflammatory conditions [47]. Under our experimental condition, we were able to show that both UV and DEE were able to increase NOX4 levels, suggesting that beside the exogenous increase of ROS due to the UV and DEE mechanism, there is also an aberrant endogenous production of ROS that can further perpetuate the damage. Furthermore, our results confirm that ROS‐dependent NOX4 activation, as it was prevented by the topical application of the antioxidant mix.

Regarding the topical formulation used in our investigation, it is composed of vitamins C and E stabilised by a powerful plant antioxidant, ferulic acid, a phenolic compound able to interact synergistically with L‐ascorbic acid and α‐tocopherol, acting as a sacrificial substrate [39]. The potent antioxidant potential of ferulic acid relies on the ability to react with reactive radicals by readily forming a resonance stabilised phenoxy radical, unable to initiate or propagate a radical chain reaction [48]. Therefore, ferulic acid protects membranes from lipid peroxidation and neutralises alkoxyl and peroxyl radicals [49]; this could explain the efficiency of the antioxidant mix used in this study in preventing 4HNE protein adduct formation. Finally, it has been demonstrated that ferulic acid is able to quench oxidative stress also by inducing the activation of the NRF2 pathway, the master regulator for the endogenous antioxidant defence. Mahmoud AM, *Environ Sci Pollut Int* 2020. It should also be considered that vitamin E is able to prevent lipid peroxidation by scavenging lipid peroxyl radicals and to break the chain propagation independent of the type of free radicals that induce chain initiation. Vitamin E is also a neutralising agent for peroxyl radicals to form tocopheryl radicals, which can be regenerated by L‐ascorbic acid [50]. In addition, ferulic acid scavenged hydroxyl radicals and superoxide radicals [39]. Therefore, the pleiotropic properties of this antioxidant mix could explain its good efficiency in preventing the formation of lipid peroxidation molecules after the pollutant insult. We confirmed that this topical application could also prevent oxidative damage from other outdoor stressors besides UV light, such as DEE, a phenomenon already demonstrated in skin tissue exposed to this pollutant, both alone and in combination with UV and O_3_ [16, 51].

Increased production of ROS and oxidative damage lead to premature ageing of the skin, which is characterised by the extensive loss of collagen, elastin fibres, and other important proteins essential components of a healthy tissue. Collagen types I and III are interwoven to form an intra‐dermal net of long collagen fibrils. During extrinsic ageing, the skin dramatically loses Collagen I and III. The long collagen fibrils, elastic fibres, glycoproteins and glycosaminoglycans are no longer interwoven to form a functional network but form an unorganised dermal‐spreader agglomeration. As a consequence of this process, elastases and metalloproteinases are activated, leading to the degradation of the dermal extracellular matrix. This process was confirmed in our study by the fact that both Col1 and Col3 were clearly decreased after exposure to UV or DEE. Of note, the antioxidant mix prevented this effect. A similar pathophysiological process was observed for tropoelastin and elastin, whose levels were significantly influenced by the two pollutants but protected by the topical antioxidant mix, confirming that the formation of ROS is a key step in premature skin ageing induced by external factors. Of note, the antioxidant mix used in the present study was able to maintain the physiological levels of elastin and tropoelastin and did not have any effect at the transcriptional levels, suggesting that its effect is mainly due to preventing oxidative damage rather than stimulating new synthesis.

As previously mentioned, UVA penetrates the skin more deeply than UVB, reaching the epidermal stem‐cell‐rich basal layer and the dermal layer. In the dermis, UVA activates matrix metalloproteinases which, in turn, degrade collagen and elastic fibres, leading to the characteristic thin, sagging, wrinkled and crepe‐like appearance of photodamaged skin [52, 53]. Therefore, being able to prevent this cascade of events could be a key approach to avoid skin damage. In this regard, in our study, the use of the antioxidant mix can maintain at basal levels the expression of MMP9 that, instead, was induced in the skin after exposure to both UV rays and DEE. In line with this result, a clear protective effect towards collagen and elastin fibres was also highlighted in our experimental conditions.

As it concerns the PM, the skin is able to absorb the organic and inorganic compounds such as PAHs and metals attached to the particles, and this can lead not only to an oxinflammatory cascade but also to the disturbance of the skin barrier [8, 32, 54]. In a previous study, we were able to show that the combined effect of UV, O_3_ and DEE was able to activate the aryl hydrocarbon receptor (AhR) [16]. This receptor is involved in recognising the xenobiotic compounds, and when activated, it leads to the transcription of cytochrome P4501A1 that can increase ROS levels and induce oxidative damage [55]. Therefore, through multiple mechanisms, both UV and DEE can alter the oxinflammatory skin state that can then lead to a compromised skin barrier [56]. This effect was confirmed by the loss of key structural proteins involved in maintaining barrier functions such as filaggrin, loricrin, claudin‐1 and desmocollin‐1 [57]. As components of the physical barrier represented by the outermost layer of the skin, these proteins are essential to confer the skin protection against trans‐epidermal water loss and penetration from external contaminants and microbes [57]. In this regard, by altering the ability of the skin to prevent the entrance of noxious compounds, PM and UV can promote positive feedback where more external insults can enter into the skin and further augment the damage. Here, the topical application of the antioxidant mix also confirmed to protect the skin barrier function, preventing damage induced by external stressors and suggesting that barrier disruption, directly or indirectly, is also driven by oxidative stress.

In the dermatological field, it is now clear that environmental insults such as UV light and atmospheric pollutants can have additive or even synergistic effects [58, 59]. Here, we focused our investigation on better elucidating the specific tissue responses triggered by UV radiation and PM, comparing their effects on skin tissue rather than their combined action. Our interest was to highlight the different effects triggered by these two pollutants with the aim of developing targeted strategies to prevent their harmful properties. Indeed, with the global increase in air pollution and as it is impossible to escape from the pollutome phenomenon [60], it becomes essential to find effective approaches to strengthen our skin barrier, delaying premature skin ageing and preventing cutaneous disorders. A two‐pronged approach that combines topical applications and dietary interventions represents the optimal option to achieve this goal [61, 62]. There is substantial evidence suggesting that a balanced diet enriched with plant‐based foods and oral supplements with bioactive compounds naturally supports healthy skin. However, although the dermis is a highly vascularized system that can directly benefit from bioactives introduced through the diet, the epidermis does not contain blood vessels and can only receive oxygen and nutrients from the blood through diffusion or absorption. Furthermore, being the outermost layer of the skin, the epidermis is more susceptible to the harmful effects of exposome factors and it is strongly suggested to apply topical antioxidants to prevent skin damage. In fact, the gradient of antioxidants present in the epidermis is characterised by higher concentrations towards the basal layers and lower concentrations in the upper layers [63], suggesting that endogenous antioxidants are continuously dissipated by external insults. This is an important aspect to take into consideration as it highlights the crucial role of the topical applications of antioxidant compounds to further enrich our natural defences as demonstrated in our study.

Finally, it should be mentioned that different pollutants can interact and lead to possible further damage. Recently, it has been shown the ability of UV to activate the PAH present in the PM, resulting in the production of derivatives with hydroxyl and ketone groups that can themselves be further activated by UV and lead to more damaging molecules including ROS. This process has been suggested by Marrot and defined as cutaneous photo‐pollution. Marrot L. *Current Medicinal Chemistry*, 2018, 25, 5469.

Therefore, it is important not only to define and compare the effect of different pollutants but also to evaluate their possible additive or even synergetic effect. This second part is possible only once we have well defined the single pollutant mechanism of action so as to clarify whether there is a possible combined damage.

## Author Contributions

The authors take full responsibility for this article.

## Conflicts of Interest

The authors declare no conflicts of interest.

## Supporting information


**Figure S1.** H&E staining of human skin explants exposed to DEE or UV light and pre‐treated with AOX mix at two‐time points, that is, Day 1 and Day 4.

## Data Availability

The data that support the findings of this study are available from the corresponding author upon reasonable request.
