# Supplementary figures and images for: Comparing UV and Diesel Cutaneous Damage and Evaluating the Protective Role of a Topical Antioxidant Mixture Containing Vitamin C, E and Ferulic Acid
Source: Exp Dermatol. 2025 Mar 10;34(3):e70069. doi: 10.1111/exd.70069 (PMC11891958; doi:10.1111/exd.70069)

## Slide 1
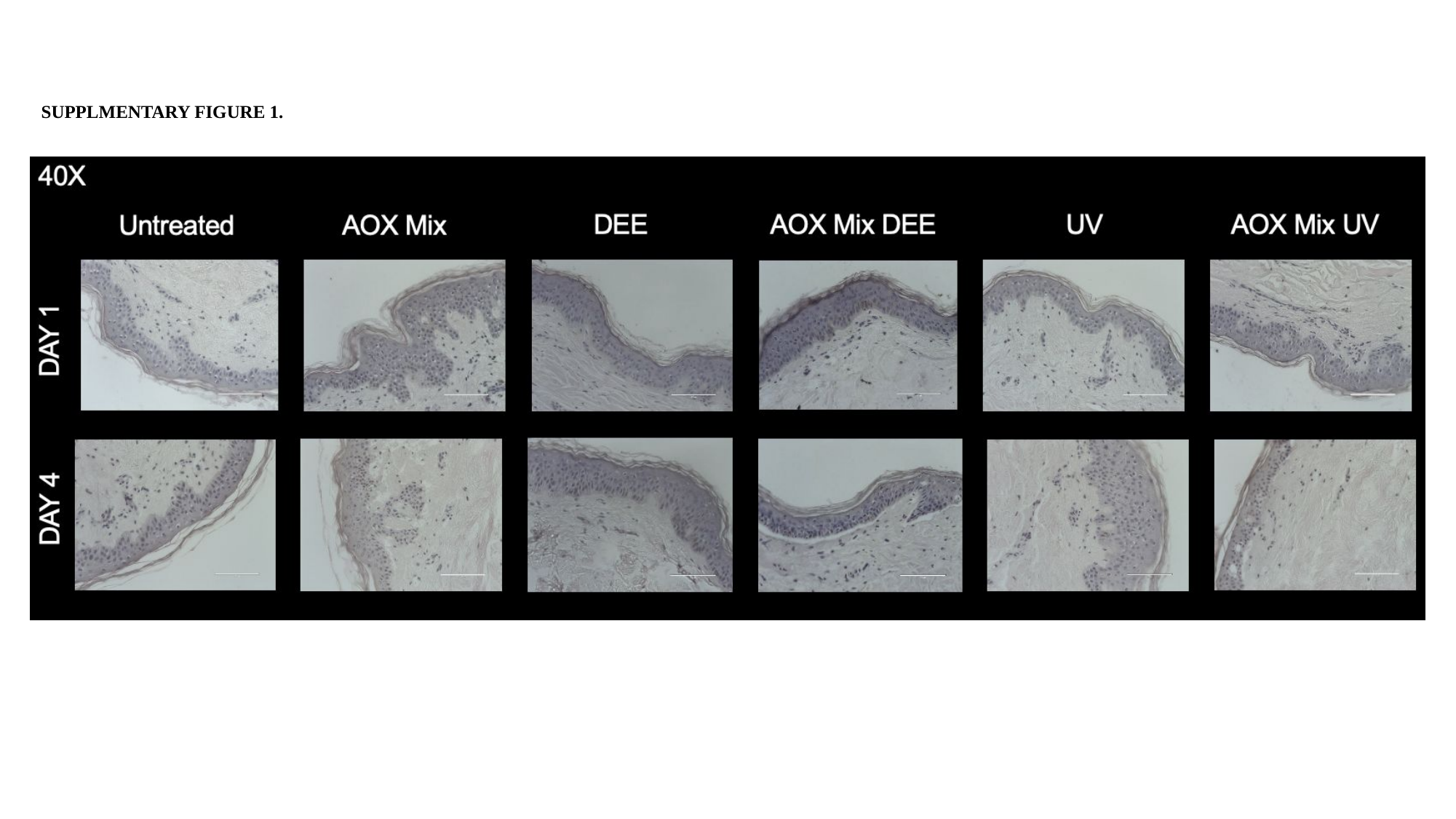

SUPPLMENTARY FIGURE 1.

Supplement: Supplementary file 1 — Figure S1. H&E staining of human skin explants exposed to DEE or UV light and pre‐treated with AOX mix at two‐time points, that is, Day 1 and Day 4. [file EXD-34-e70069-s001.pptx]
